# Active Surveillance in Metastatic Renal Cell Carcinoma

**DOI:** 10.15586/jkcvhl.v11i2.309

**Published:** 2024-06-04

**Authors:** Nicholas Beecroft, Timothy D. Gauntner, Rituraj Upadhyay, Shang-Jui Wang, Yuanquan Yang, Eric A Singer, Shawn Dason

**Affiliations:** 1 Division of Urologic Oncology, The Ohio State University Comprehensive Cancer Center, Columbus, OH;; 2 Division of Medical Oncology, The Ohio State University Comprehensive Cancer Center, Columbus, OH;; 3 Department of Radiation Oncology, The Ohio State University Comprehensive Cancer Center, Columbus, OH

**Keywords:** active surveillance, cytoreductive nephrectomy, metastatic renal cell carcinoma, radiation therapy, systemic therapy

## Abstract

Metastatic renal cell carcinoma (mRCC) is a heterogenous disease with a variable clinical course. While therapies for treatment of this condition have progressed, they are not without toxicity. In some patients, active surveillance (AS) of this disease is increasingly considered to delay its toxicity. This article seeks to review the literature and discuss management of metastatic renal cell carcinoma, specifically regarding upfront AS, the role of radiation therapy in delaying systemic therapy, and surveillance after initial treatment with systemic therapy. Median time on AS prior to initiation of systemic therapy ranged from 14 to 60 months across studies. AS is appropriate to offer in favorable or intermediate risk, asymptomatic, and systemic treatment naïve patients with mRCC.

## Introduction

Renal cell carcinoma (RCC) is the seventh most common malignancy in the United States, with an estimated 82,000 new cases annually (1). The metastatic renal cell carcinoma (mRCC) population consists of the patients with synchronous and metachronous metastases. Synchronous metastases are present in one-third of patients newly diagnosed with RCC. Meanwhile distant metastases present metachronously in about 30% of those undergoing nephrectomy for localized RCC (pT1-3, N0 at the time of nephrectomy) (2).

The clinical course of mRCC can vary significantly. The most commonly used prognostication tools are the International Metastatic Renal Cell Carcinoma Database Consortium (IMDC) and the Memorial Sloan Kettering Cancer Center Criteria (MSKCC) risk models (3, 4). Using these models, patients can be stratified into good risk (0 factors), intermediate risk (1–2 factors), and poor risk (>2 factors) groups to estimate survival (Table 1).

**Table 1:** The International Metastatic Renal Cell Carcinoma Database Consortium (IMDC) and the Memorial Sloan Kettering Cancer Center Criteria (MSKCC) risk model-included prognostic factors.

Included Prognostic Factors	IMDC	MSKCC
Karnofsky performance < 80%	✓	✓
Time from diagnosis to treatment <1 year	✓	✓
Hemogloblin < lower limit of normal	✓	✓
Corrected calcium > upper limit of normal	✓	✓
Neutrophil > upper limit of normal	✓	✘
Platelet > upper limit of normal	✓	✘
Lactate dehydrogenase (LDH) > 1.5× the upper limit of normal	✘	✓

Systemic therapy (ST) for mRCC has markedly improved survival in recent decades. The current standard for the first-line ST for most patients with mRCC is a combination therapy with two immune checkpoint inhibitors (IO) or an IO and a tyrosine kinase inhibitor (TKI) (5). While generally well-tolerated, toxicities associated with these modern ST regimens may impact quality of life (QoL). Grade-3 adverse events or higher have been reported in 46–83% patients undergoing IO–IO or IO–TKI regimens (6–9).

**Figure 1:**
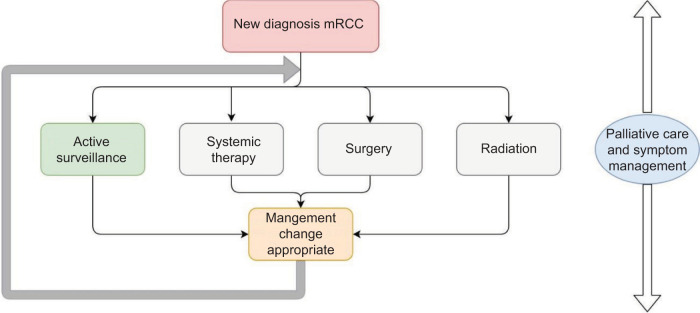
mRCC treatment algorithm.

Active surveillance (AS) in mRCC is increasingly considered to delay the toxicities of ST. AS is defined as observation of known mRCC without active treatment (5). Patients undergo AS with the intent of receiving life-prolonging treatment upon progression. AS can be the primary strategy upon diagnosis of mRCC or it can follow cytoreductive nephrectomy, metastasectomy, stereotactic ablative radiotherapy (SABR), or ST (Figure 1). AS may involve radiologically evident disease or may be conducted in patients with no evidence of disease (NED) following other treatments. Current guidelines from the National Comprehensive Cancer Network (NCCN), the American Society of

Clinical Oncology (ASCO), and European Association of Urology are summarized in Table 3 (5, 10, 11). Our objective in this review is to discuss the supporting evidence and practical application of AS across the spectrum of mRCC management.

**Table 2:** Studies examining AS in mRCC population.

Study	Design	Number of patients included in analysis	Population characteristics	Median follow-up	Median time on AS prior to ST	Median overall survival	Surveillance plan
Rini et al. (12)	Prospective, single-arm, multicenter	48	IMDC risk: Favorable: 23% Intermediate: 75% Poor: 2% 47/48 patients had nephrectomy prior to study Tumor burden baseline 3.2 cm	38 months	14.9 months	Median OS not reached, estimated 44.5 months	CT CAP: Q3 months 1st year Q4 months 2nd year Q6 indefinite Brain and bone scan within 12 months of baseline, then only if symptoms are present Recommended yearly CNS imaging after study, given development of metastases in two patients on AS
Harrison et al. (13)	Prospective, double arm, multicenter	AS: 143 ST: 305	31% of AS cohort had no visible metastasis at start of study 56% of patients had a prior nephrectomy IMDC risk (AS/ST groups): Favorable: 60%/14% Intermediate: 38%/65% Poor: 2%/22%	AS: 33 months	60 months (including those with prior diagnosis of metastasis but currently NED)	Median OS not reached Estimated 122 months in those with metastatic disease on imaging	No specific schedule
Kushnir et al. (14)	Retrospective, multicenter, 3 cohorts (A: AS, B: upfront ST, and C: death within 1 year prior to ST)	A: 853 B: 827 C: 119	A: 54% underwent nephrectomy B: 45% underwent nephrectomy IMDC risk (A/B): Favorable: 26%/8.6% Intermediate: 58%/57% Poor: 15.8%/34.5%	56.7 months	14.2 months (in 364 patients who started ST)	Median OS not reached for AS group; 5-year OS probability was 70%	No specific schedule
Matsubara et al. (15)	Retrospective, single-institution, single-AS cohort	29	100% underwent nephrectomy MSKCC risk: Favorable: 62% Intermediate: 38% Tumor burden baseline: 3.5 cm	35.4 months	Median progression-free survival was 26.1 months, only 58% of patients received systemic treatment after progression	Median OS not reached; 84% survival at 48 months	Initial monthly examinations followed by Q3 month examinations with laboratories CT CAP Q3–Q4 months Brain imaging and bone scans at clinician discretion
Park et al. (16)	Retrospective, single-institution, single-AS cohort	58	97% had prior nephrectomy MSKCC risk: Favorable: 29% Intermediate: 59% Poor: 2%	31.4 months	20 months	Median OS not reached; estimated 91 months	Radiographic images Q6–Q8 weeks for 6 months then Q8–Q12 weeks for the next 6 months, Q12 weeks from years 1 to 3, then from Q16 to Q24 weeks indefinitely
Bimbatti et al. (17)	Retrospective, multicenter, single-AS cohort	52	98% had prior nephrectomy Tumor burden baseline 2 cm IMDC risk: Favorable: 69% Intermediate: 25% Poor: 6%	38.5 months	18.3 months	Median OS not reached; estimated 80.1 months	At least 1 CT scan Q6 months for the first 4 years and then Q1 year thereafter
Stares et al. (18)	Retrospective, single-Institution, single-AS cohort	160	92.5% underwent nephrectomy: 58% initial curative intent, 34% cytoreductive IMDC risk: Favorable: 29% Intermediate: 60% Poor: 11%	49.6 months	Time from diagnosis of mRCC to initiation of ST: 31.8 months	Median OS not reached; estimated 88 months	Radiologic and clinical review Q3–Q4 months initially in oncology clinic

Abbreviations. IMDC: International Metastatic Renal Cell Carcinoma Database Consortium; OS: overall survival; CT CAP: computed tomography chest abdomen and pelvis; CNS: central nervous system; AS: active surveillance; ST: systemic therapy; NED: no evidence of disease; MSKCC: Memorial Sloan Kettering Cancer Center Criteria.

**Table 3:** North American and European AS mRCC guidelines.

Guideline	AS recommendations
NCCN (10)	Listed as useful in certain circumstances as first line in the favorable risk-clear cell histology population.
ASCO (5)	Appropriate in selected patients with clear cell mRCC. Patients include those with IMDC favorable and intermediate risk, limited or no disease-related symptoms (DRS), favorable histologic profile, a long interval between nephrectomy and development of metastasis, or with limited metastatic burden.
EAU (11)	Observation of oligometastatic disease recurrence is mentioned as common in real world settings after ruling out rapid progression.

## Body

### Methods

The National Institutes of Health’s PubMed was used to identify English language articles from 2005 to 2023 pertaining to the use of AS and other management modalities in the mRCC population. Search terms included “metastatic renal cell carcinoma,” “advanced renal cell carcinoma,” “outcomes after systemic therapy,” “active surveillance,” and “radiation therapy.” Current North American and European mRCC guidelines were reviewed.

### Patient population

Across the studies included in this review, median age was 60–70 years. About three-quarters of patients were males. Most patients had metastasis to 1 or 2 organ systems and underwent nephrectomy with curative intent or cytoreductive nephrectomy. The vast majority were either favorable or intermediate risk using IMDC or MSKCC criteria. When selecting patients in a real-world setting, those with IMDC favorable and intermediate risk, few to no symptoms related to their disease, favorable histologic profile (low-grade or absence of sarcomatoid features), interval of more than 1 year between nephrectomy and development of metastasis, and/or those with limited burden of metastatic disease were considered (12, 13).

### Upfront Active Surveillance

Upfront AS refers to those managed with AS prior to receiving ST for mRCC. There are two contemporary prospective studies examining upfront AS in mRCC patients. In addition to the two prospective studies, our review identified five retrospective studies examining this management strategy, as summarized in Table 2 (12–18).

Median time of surveillance ranged from 14.2 to 60.0 months. Study inclusion criteria were heterogeneous—a prolonged median time on surveillance was evident in studies that included those with a diagnosis of mRCC but no current visible disease on imaging. Estimated median overall survival (OS) ranged from 44.5 to 122 months. A large majority of patients across studies had nephrectomy prior to the start of AS. These AS series were primarily done in the era of TKIs being the standard ST. Diagnosis of disease progression generally prompted initiation of ST.

### Role of Radiation Therapy in Delaying Systemic Therapy for mRCCs

Stereotactic ablative radiation therapy (SABR) has emerged as an effective treatment modality for mRCC patients who were traditionally considered relatively radio-resistant. SABR allows precise delivery of a high dose of radiation per fraction in up to 1–5 fractions. SABR is safe and effective offering >90% local control (LC) of mRCC at 1 year (19–21). Given the noninvasive nature of SABR, it is increasingly used in the management paradigm of mRCC, as it can provide excellent local control in patients with limited metastatic burden to delay the initiation of ST (22).

Several recent retrospective as well as prospective studies have evaluated the role of metastasis-directed therapy with SABR in delaying ST in patients with ST naïve oligometastatic RCC (23). Hannan et al. prospectively evaluated this strategy in patients with ≤3 extracranial metastases, with the primary end-point of delay in ST by >1 year in at least 60% of the patients (24). In 33 patients with 57 treated sites, freedom from systemic therapy (FFST) at 1 year was 91.3%, well exceeding the endpoint. The authors observed a 1-year overall survival of 95.7%, compared to contemporary studies. In this study, none of the patients had grade 3 or higher toxicities, and QoL was largely unaffected. Lastly, the median time to systemic therapy (TTST) in this study was 17.1 months, compared to 14.9 months on AS in the study conducted by Rini et al. (12). In another phase II study, Tang et al. investigated the efficacy of definitive-intent radiation therapy (RT) to all metastases in 30 oligometastatic RCC patients (with up to five sites of metastatic disease) (25). At median follow-up of 17.5 months, the trial showed impressive 1-year progression-free survival (PFS) and FFST of 64% and 82%, respectively. About 10% of patients had grade 3 or higher adverse events, such as grade-3 back pain, grade-3 muscle weakness, and grade-4 hyperglycemia in one patient each.

Key advantages of upfront SABR approach are low toxicity rates and ability to preserve QoL by postponing the start of ST. How best to select patients for SABR to delay ST is unclear but likely includes patients with good performance status and limited (up to 5) sites of metastatic disease. Future prospective studies comparing upfront SABR to observation could shed more light on clinical outcomes and biomarkers for appropriate patient selection.

### Surveillance after Systemic Therapy for mRCC

The approval of combination IO–IO and IO–TKI regimens (Table 4) has improved OS and durability of responses over single agent TKI in first-line ST for mRCC (6–9, 26, 27). Patients typically discontinue ST after achieving a complete response (CR), encountering treatment-limiting toxicity, or completing the planned IO course (although patients on IO–TKI regimens typically continue TKI after completing planned IO). Among the patients who discontinue ST due to toxicity or completion of planned IO, many remain treatment-free for substantial period despite having active disease (28). This population—patients who have stopped all therapies after first-line ST but still have active disease—is appropriate for AS, with follow-up imaging recommended every 6–16 weeks as per NCCN guidelines (10). Detailed guidelines for AS in this context are lacking—largely because most phase-3 follow-up studies have not reported outcomes for this specific patient population. However, surveillance strategies can be described by general outcomes data of IO–IO and IO–TKI clinical trials.

**Table 4:** Summary of pivotal phase 3 trials evaluating combination treatments for metastatic ccRCC.

Phase 3 trial	Treatment design	# Pts in experimental ITT arm	CR rates in experimental ITT	Reported metrics for patients who completed 2 years of IO	TRAEs leading to stoppage of ≥1 drug in combo arm, n (%)	Reported metrics for patients who discontinued (DC) ≥1 drug due to TRAE
CheckMate 214 (6, 44)	IO/IOvs TKI-randomized patients to Ipi/Nivo or Sun	550	11.6% at 5-year follow-up	75% of patients in Ipi/Nivo arm discontinued treatment by ~22 months	127 (23)	OS at 24 months was 74% for patients who discontinued Ipi/Nivo vs 61% for those who discontinued Sun
KEYNOTE-426 (9)	IO/TKI vs TKI-randomized patients to pembro/axi or Sun	432	11.6% at 5-year follow-up	29.9% of IO/TKI cohort completed 2 years therapy; PFS 32.8% at 60-month & CR 18.3% in this subgroup	111 (25.9)	Not reported
CheckMate 9ER (8)	IO/TKI vs TKI-randomized patients to Nivo/Cabo or sunitinib	323	13% at 3-year follow-up	36% of IO/TKI arm completed 2 years of NIVO; median time to next-line treatment or death at 20.6 months	87 (27)	Not reported
CLEAR (7)	IO/TKI vs TKI/mTORi vs TKI-randomized patients to Len/Pembro, Len + everolimus or Sun	355 (Len + Pembro)	18.3% at 4-year follow-up	28% of IO/TKI arm completed 2 years of Pembro; OS 94.5%, with 68.3% experiencing TRAEs in this subgroup	131 (37.2)	Not reported
IM motion 151 (27)	IO/Bev vs TKI-randomized patients to Atezo/Bev or Sun	454	9%	12% of IO/Bev arm remained on Atezo at 5-year (40 months min) follow-up	128 (28)	Not reported
JAVELIN Renal 101 (26, 45)	IO/TKI vs TKI-randomized patients to avelumab/axi or Sun	442	3.8% at 34.1-month median follow-up	NR	138 (31.8%)	Median OS & PFS were 29.8 and 11.1 months for patients who discontinued avelumab/axi vs 37.8/14.0 months for patients who discontinued Sun
COSMIC 313 (46)	IO/IO/TKI vs IO/IO-randomized patients to Ipi/Nivo/Cabo or Ipi/Nivo	428	3% at median 17.7-month median follow-up	NR	193 (45)	Not reported

Abbreviations. IO: immune-checkpoint inhibitors; TKI: tyrosine kinase inhibitors; CR: complete response; TRAE: treatment- or immune-related adverse events; Ipi: ipilimumab; Nivo: nivolumab; Atezo: atezolizumab; Pembro: pembrolizumab; Axi: axitinib; Len: Lenvatinib; Cabo: cabozantinib; Sun: sunitinib; Bev: bevacizumab; PFS: progression-free survival; mTORi: mammalian target of rapamycin inhibitor; NR: not reported.

Immune-related adverse events (TRAE) often correlate with IO responses (Table 4); these are durable in a subset of patients, including those who have only achieved a partial response to therapy. The best evidence is from CheckMate 214, where 23% of patients discontinued ipilimumab/nivolumab (ipi/nivo) due to TRAE (29). At a minimum 30-month follow-up, 42% patients who discontinued ipi/nivo due to TRAE remained treatment-free at 24 months and 12% had complete response (28). In the JAVELIN renal 101 trial, patients in the avelumab/axitinib arm, who came off the study due to TRAE but received no second-line therapy, had a median overall survival of 21.3 months (30). HCRN GU16 260, a phase-II study of nivolumab and salvage ipi/nivo for treatment-naïve patients with mRCC, was designed to reduce toxicity by capping nivo therapy at 2 years while providing salvage ipi/nivo at the time of disease progression or for stable disease at 48 weeks (31). A 36-month follow-up of 128 patients from this trial showed a mean treatment-free survival of 9.4 months, with 38.5% of patients alive and subsequently treatment-free (32). Indeed, patients who stop IO due to TRAE appear to be enriched for responders with a potential for extended treatment-free intervals necessitating AS even in the absence of a complete response.

Most of the phase-3 IO–TKI studies maintained patients on IO for up to 2 years and continued TKIs until disease progression or unacceptable toxicity (6–8). While data of specific outcomes for patients who completed planned IO and then stopped TKI due to toxicity are unreported, data from a TKI interruption study suggest that outcomes of IO completers who continue TKI might approximate those in AS cohorts who have discontinued all ST (Table 4) (33). For instance, an analysis of 129 patients (29.9% of IO–TKI cohort) in the KEYNOTE-426 trial who completed 2 years of pembro showed a PFS of 55.2% at 36 months and 32.8% of PFS at 60 months (34, 35).

Finally, a systematic review of trials evaluating responders to IO–IO, IO–TKI, or single agent IO in first- and second-line settings for mRCC discovered mean pooled treatment-free survival rates of 35% and 20% at 6 and 12 months, respectively, for responders who discontinued IO due to various reasons (36). Overall, these data highlight the potential for responders to remain without therapy for a considerable duration of time before disease progression.

## Discussion

Increasing evidence is discovered that upfront AS is a viable option for patients with mRCC. Median time on AS has ranged from 14 to 33 months prior to the start of ST in the discussed studies. Furthermore, median overall survival is reassuring in the AS cohorts of two prospective studies: 44 months in Rini et al.’s series, and not reached in the 33 months of follow-up in Harrison et al.’s report (12, 13).

### Predictors of length of active surveillance

Predicting length of remaining AS is challenging. Several factors have been proposed to be predictors of time on AS in the published literature (Table 5). Rini et al. had published data on whole exome sequencing and RNA sequencing on tumors for 37 of their 48 patients. The authors found that on multivariable analysis, presence of *TP53* and *SMARCA4* mutations was associated with shorter time on AS (37). Nizam et al. after their review of the literature emphasized that the presence of systemic symptoms, such as fevers, chills, and night sweats, potentially indicated that patients would benefit from ST, rather than a period of AS or upfront cytoreductive nephrectomy (38). Broadly speaking, patients with low metastatic disease burden and other positive risk factors seem to do better on upfront AS, but reproducible prognostic biomarkers for better risk stratification remain elusive. Disease biology is also certainly an important consideration while determining surveillance strategies for patients who have discontinued first- or second-line therapy for mRCC due to either complete response or toxicity. The underlying tumor biology in mRCC that has progressed after an initial response to ST is undoubtedly different from that of untreated synchronous or metachronous metastases. Therefore, follow-up or surveillance strategies for disease arising after an initial favorable response to ST require prospective evaluations.

**Table 5:** Predictors of length on AS.

Study	Prognostic Factors
Rini et al. (12)	Age, gender, KPS, time from diagnosis to metastatic disease, number of IMDC risk factors, IMDC prognostic group, number of MSKCC risk factors, MSKCC prognostic group, number of metastatic sites, baseline tumor burden, presence of lung, non-lung, or both lung and other organ metastases were investigated. IMDC risk factors and number of metastatic sites were found to be prognostic for the length of AS. Proposed favorable risk group made up of patients with 0–1 IMDC risk factors and 2 or fewer organs with metastases. Unfavorable risk group, including all other patients. (Rini classification). Subsequent whole exome and RNA tumor sequencing was done. Presence of *TP53* and *SMARCA4* mutations were associated with shorter time on AS.
Harrison et al. (13)	IMDC risk group and time from initial diagnosis to the diagnosis of metastases <1 year were investigated. Both were found to be predictive of OS for the ST cohort but not for AS cohort.
Matsubara et al. (15)	Patient gender, histology (cc vs non-ccRCC), number of metastatic sites, albumin, hemoglobin, LDH, calcium, CRP, and disease status (synchronous vs metachronous metastases) were investigated. Only disease status was found to be statistically significant regarding 2-year overall survival. No parameters were significant for progression free survival.
Park et al. (16)	Neutrophilia, thrombocytosis, KPS, presence of liver metastases, time from diagnosis to AS <1 year, poor Heng risk group, and histology (cc vs non-ccRCC) were investigated. On multivariable analysis, KPS, presence of liver metastasis, and time from diagnosis to AS <1 year were found to be predictive for worse time to progression.
Bimbatti et al. (17)	Number of metastatic sites, Rini classification, TB (sum in millimeters of the longest tumor diameter of each measurable lesion), and IMDC classification were investigated. Only initial IMDC classification was found to be predictive for time on AS. During AS, increased number of metastatic sites and increase in TB were negative prognostic factors for OS.
Stares et al. (18)	Hemoglobin, WBC, neutrophil count, platelet, calcium, albumin, CRP, modified Glasgow prognostic score, Fuhrman grade, presence of necrosis, number of IMDC risk factors, IMDC risk group, age, gender, number of organs involved, Rini classification, presence of lung, bone, adrenal, lymph metastases, histology (cc vs non-ccRCC), and time from initial diagnosis to metastatic disease were investigated. On multivariable analysis, only CRP >10 mg/L was found to be associated with shorter time on AS. CRP and presence of lymph node metastases were independently predictive of OS.

Abbreviations. KPS: Karnofsky Performance Status; IMDC: International Metastatic Renal Cell Carcinoma Database Consortium; MSKCC: Memorial Sloan Kettering Cancer Center Criteria; OS: overall survival; AS: active surveillance; ST: systemic therapy; LDH: lactate dehydrogenase; CRP: C-reactive protein; TB: tumor burden; WBC: white blood cell count; cc: clear cell; ccRCC: clear cell renal cell carcinoma.

### Non-clear cell RCC

The applicability of AS to non-clear cell mRCC is uncertain. Non-clear cell mRCC patients are included in both prospective and retrospective trials but remain a small proportion of studied patients, ranging from 4% to 24%. Histology is investigated as a prognostic marker in multiple studies but not discovered as a predictive of time on AS or overall survival. As such, it seems reasonable to offer AS as a treatment option for non-clear cell RCC patients, with the knowledge that there is less data to support its use in rarer RCC subtypes.

### Surveillance protocol

A uniform surveillance protocol is yet to be developed in patients with a history mRCC appropriate for AS. The protocols described by studies are listed in Table 2. Rini et al. followed a stricter protocol with slowly spaced-out computed tomography (CT) imaging (12). Initially central nervous system (CNS) imaging was at providers’ discretion; given the two patients who developed symptomatic CNS disease, they recommend at least yearly CNS imaging. This appears reasonable, given that the new CNS metastases on AS protocol was described in the Park et al. series as well (16). See Table 6 for proposed AS protocol.

**Table 6:** Proposed AS protocol.

	Baseline	0–12 months from start of AS	12–24 months from start of AS	24 months onward from start of AS
CT chest abdomen, and pelvis	Recommended	Every 3 months	Every 4 months	Every 6 months
CT or MRI brain	Recommended	At 12 months	At 24 months	Every 12 months
Bone scan	Recommended	As indicated clinically	As indicated clinically	As indicated clinically
Chemistry and complete blood count	Recommended	Every 3 months	Every 4 months	Every 6 months

Abbreviations. AS: active surveillance; CT scan: computed tomography scan; MRI: magnetic resonance imaging.

### Prior nephrectomy

Across studies, most patients had prior nephrectomy (54–100% of patients). Many patients underwent a nephrectomy with curative intent and later presented with metachronous recurrence. As late metachronous recurrences generally have lower disease burden and higher proportion of favorable risk patients, these patients are probably be good AS candidates. Some patients presenting with synchronous mRCC are treated with cytoreductive nephrectomy, a highly nuanced decision (39). As cytoreductive nephrectomy is typically done to control the bulkiest and most symptomatic disease burden, those eligible for cytoreductive nephrectomy are also considered for AS. Roussel et al. reported in their retrospective series of 119 patients that those who were eligible for a period of AS following cytoreductive nephrectomy had a greater overall survival (56 months), compared to those who required upfront TKI only (13 months) or cytoreductive nephrectomy immediately followed by TKI (17 months) (40).

### Defining progression

In all described studies, patients underwent regular diagnostic imaging to monitor progression of the disease. When reported, disease progression was determined using Response Evaluation Criteria in Solid Tumors criteria (RECIST) (41). However, radiographic evidence of progression did not always prompt termination of AS. For example, in Rini et al.’s cohort, 53% of those with progressive disease immediately started ST and 47% continued on surveillance for a time(12). Timing of initiation of ST is a nuanced shared decision between patient and oncologist and not solely determined by RECIST progression.

### Salvage treatment

Although the period of AS varies, most patients require salvage therapy at some point due to radiographic progression or development of symptomatic metastases. For the studies discussed, patients typically received TKIs as ST. AS patients generally respond well to ST; Kushnir et al. found that time to systemic treatment failure (defined as cessation of first-line ST due to progression, toxicity, or death) was longer in their AS cohort, compared to their upfront ST cohort (12.6 vs 8.1 months), suggesting that it is safe to wait to begin ST (14). Furthermore, some evidence demonstrated that a delay in starting the treatment in patients who were planned to undergo ST did not substantially impact outcomes. Iacovelli et al. reported outcomes comprising 635 patients planned for treatment with TKI (42). The authors found that treatment delay was common and the median period was 6.3 weeks. When stratifying patients into those who were delayed for >6 weeks and those <6 weeks, they found no difference in median PFS and overall survival. However, these patients required careful monitoring—in Stares et al.’s cohort, six of 160 patients on AS developed rapid progression and died without receiving ST, as they were no longer fit enough to tolerate the therapy (18). As with any monitored cancer, there is always a risk that it progresses without treatment. Further studies are needed to quantify this risk more definitively.

### Quality of Life

Avoiding the negative effects of ST is a driver for patients to consider AS. Both prospective studies collected QoL data (12, 13). Harrison et al.’s cohort reported QoL in the form of the Functional Assessment of Cancer Therapy–Kidney Symptom Index (FACT-FKSI) and the FACT-General questionnaires (FACT-G). Scores were significantly higher in the AS cohort than the ST cohort, suggesting a higher QoL. Rini et al. (12) assessed QoL using the FACT-FKSI and the Hospital Anxiety and Depression Scale (HADS) at baseline as well as with each CT scan. Scores consistent with anxiety were identified on 16% of patients on FKSI–Disease-*Related Symptoms* (DRS), and scores consistent with depression on the HADS questionnaire. No significant changes were observed in patients on surveillance, suggesting that AS is well tolerated over time despite the probable progression in some of such patients. To compare, in one series of patients with mRCC receiving ST, 31% had depression prior to starting ST, which increased to 43% after 12 weeks of therapy (43).

## Future Perspective

As more data emerge, AS is increasingly discussed as a viable management option for appropriate patients. Further prospective studies would allow better prediction about patients succeeding with AS. Hopefully, the work done thus far with prognostic biomarkers would be built on until decision guiding biomarkers are available for determining AS.

## Conclusion

Upfront AS should be discussed with favorable and intermediate risk in asymptomatic, systemic treatment naïve patients with mRCC. Further, large and prospective studies are needed to better characterize the risks associated with delaying systemic treatment, determining prognostic biomarkers to help guide treatment decisions, and what role radiation or surgery could play for patients with oligoprogression.
